# People underestimate their capability to motivate themselves without performance-based extrinsic incentives

**DOI:** 10.1007/s11031-022-09996-5

**Published:** 2022-12-28

**Authors:** Kei Kuratomi, Laura Johnsen, Shinji Kitagami, Aya Hatano, Kou Murayama

**Affiliations:** 1 Faculty of Education, Gifu Shotoku Gakuen University, 1-1, Takakuwanishi, Yanaizu-Cho, Gifu, Japan; 2 Community Based Neurorehabilitation Team, Berkshire Healthcare NHS Foundation Trust, London Road, Thatcham, RG18 3AS UK; 3 Department of Cognitive and Psychological Sciences, Graduate School of Informatics, Nagoya University, Furo-cho, Chikusa-ku, Nagoya, Japan; 4 IdeaLab Inc., Ebisu, Shibuya-ku, Tokyo, Japan; 5 School of Information, Kochi University of Technology, 185 Miyanokuchi, Tosayamada, Kami, Japan; 6 Hector Research Institute of Education Sciences and Psychology, University of Tübingen, Europastraße 6, 72072 Tübingen, Germany; 7 Research Institute, Kochi University of Technology, 185 Miyanokuchi, Tosayamada, Kami, Japan; 8 School of Psychology and Clinical Language Sciences, University of Reading, Earley Gate, Whiteknights, Reading, RG6 6AL UK

**Keywords:** Metamotivation, Metacognition, Affective forecasting, Interest

## Abstract

Research has shown that we are endowed with a remarkable capacity to motivate ourselves in the absence of extrinsic incentives (i.e. intrinsic motivation). However, little research has been conducted to investigate whether we accurately appreciate the power of intrinsic motivation. The current research aimed to examine the metacognitive accuracy of the extent to which people can motivate themselves without performance-based extrinsic incentives. Participants were presented with a relatively long and repetitive task without extrinsic incentives, and before doing the task, they were asked to predict their motivation on completion of the task. Across seven experiments using a variety of tasks with different populations from different countries, participants were consistently engaged in the task more actively than they predicted. When participants were provided with performance-based monetary rewards, however, this bias was diminished. These results indicate that we tend to underappreciate our capability to sustain our motivation without extrinsic incentives.

**Supplementary Information:**

The online version contains supplementary material available at 10.1007/s11031-022-09996-5.

Sustaining and enhancing motivation and engagement have been long-standing issues in many applied settings such as schools and workplaces. Motivation can be influenced in diverse ways, and one major class of motivational incentives are extrinsic rewards, which refer to any incentives outside of, and instrumental to, the activity itself, such as monetary incentives (Kruglanski, 1975). However, humans are also endowed with a remarkable capacity to enjoy and be engaged in a task *without* being fueled by extrinsic rewards (Deci & Ryan, 1985; Renninger & Hidi, 2016; Murayama, 2022). Engagement and enjoyment can even be maintained in situations requiring sustained effort for relatively repetitive tasks via the use of self-regulation or self-control skills. For example, Sansone et al. (1992) showed that when participants were asked to perform necessary, but uninteresting activities (e.g., copying a letter matrix), they self-motivated themselves by generating strategies to make the task more engaging (e.g., self-setting a challenging goal).

But do we really know how motivated we are without performance-based incentives? The main purpose of the current study is to examine people’s metacognitive accuracy of motivation, especially focusing on the enjoyment and engagement aspects.^1^ Previous research has examined various factors and strategies that influence people’s self-regulation of motivation, providing important insights into how we can promote or undermine motivation (Duckworth et al., 2014; Hulleman & Harackiewicz, 2009; Thoman et al., 2007; Wolters, 2003; Yeager et al., 2014). Little research, however, has examined the metacognitive accuracy of people’s metacognitive belief about the power of motivation. This is unfortunate, as it is our metacognitive belief that guides our decisions and strategies to regulate motivation: if we have inaccurate metacognitions about our motivation, we are likely to make suboptimal decisions to regulate our own or others’ motivation (Dickson & Wendorf, 1999; Heath, 1999; MacGregor, 1960). Murayama et al., (2016a, 2016b) called such metacognitive belief or awareness about our motivation *metamotivation* (see also Scholer & Miele, 2016; Scholer et al., 2018), and pointed out the possibility that inaccurate metamotivation can lead people to adopt ineffective motivating strategies, despite their well-meaning intentions of enhancing motivation. If we underappreciate our capacity of motivation without extrinsic incentives, for example, we may end up spending more money than necessary to motivate others.

Although sparse, there have been several studies that examined people’s belief about motivation (Gurland & Glowacky, 2011; Heath, 1999; Miller & Ratner, 1998; Murayama et al., 2016a, 2016b; Woolley & Fishbach, 2015). These studies mainly focused on people’s metamotivational belief about extrinsic rewards, and they generally suggested that people tend to overestimate the motivating power of extrinsic incentives, despite potential cultural differences (DeVoe & Iyengar, 2004). None of the existent studies, however, have investigated people’s belief about their motivation in the absence of performance-based extrinsic incentives. In addition, most of these studies only examined people’s belief about motivation, and did not directly compare people’s belief with actual motivation (for an exception, see Woolley & Fishbach, 2015). This makes it difficult to evaluate the accuracy/inaccuracy of people’s metacognitive belief about how motivation works.

The current research provides empirical evidence to aid understanding of people’s metamotivational belief about motivation and its accuracy without extrinsic incentives. In seven experiments, using various tasks, with various populations, participants worked on a relatively repetitive and long task without performance-based extrinsic incentives. Critically, to examine the accuracy of participants’ metamotivation, before doing the task, participants were asked to make a prediction about their motivation at the end of the task. This predicted motivation was then compared with participants’ actual motivation, assessed after the task. We expected participants to underestimate the power of motivation without incentives—that participants would enjoy and become engaged in the task, without performance-based incentives, more actively than they predict. As indicated earlier, humans have a notable capacity to motivate and engage themselves without extrinsic incentives by producing self-motivating strategies or intrinsic rewards (Renninger & Hidi, 2016; Sansone et al., 1992). However, in a recently proposed reward-learning framework of intrinsic motivation (or interest), Murayama (2022) argued that such self-motivating mechanisms are generative and invisible in nature, and it is difficult for people to conceive of them when they make a prediction about their own motivation. As a result, participants may exhibit significant underestimation of their actual motivation.

## Experiment 1

Experiment 1 aims to provide initial evidence that people underestimate their power of motivation without extrinsic incentives. We expected that participants would show higher actual motivation (as measured by task enjoyment and engagement) than predicted motivation.

## Method

### Participants

A total of 50 UK adults were recruited online from Scientific Prolific (21 female, *M*_*age*_ = 24.4).^2^ Participants received £ 1.67 for participation. In this and the following studies, no interim statistical tests were conducted. For all studies, we predetermined sample size based on the budgetary limit and time constraints. The experiments were approved by research the Ethics Committees of University of Reading (Experiment 1, 2, 4 and 5) and Nagoya University (Experiment 3).

### Measures

In this and the following studies, we assessed participants’ motivation for the respective task focusing on the engagement and enjoyment aspects of it. We used two scales taken from Elliot and Harackiewicz (1996).^3^ Both task engagement (e.g., “I concentrated on the activity”) and task enjoyment (e.g., “I enjoy doing [task name]”) were assessed with three items on a 7-point scale (Cronbanch’s αs = 0.50–0.93). In predicted motivation and actual motivation, the correlation between task engagement and task enjoyment was 0.52 (*p* < 0.001) and 0.45 (*p* < 0.001), respectively.

### Procedure

The experiment was conducted online. Participants were instructed to work on a series of association production tasks for approximately 20 min. In this task, participants were presented with words in a random order and were asked to produce as many words as possible that were associated with the presented word. Participants typed in these words on a screen within 20 s before proceeding to the next trial. There were 60 words in total.

Participants first completed 1-min practice trials and rated their intrinsic motivation for the task (task engagement and task enjoyment) so that they gained a sense of how the task felt to complete. Before the main task, participants were asked to make a prediction about their intrinsic motivation at the end of the 20-min task (“Now you will do the same task for 20 min. Please make a prediction about how you would feel about the task in 20 min”). After the main task, participants were asked to rate their current (actual) intrinsic motivation for the task.

### Results and discussion

The comparison between the predicted motivation and actual motivation showed that participants enjoyed (*M* = 4.15, *SD* = 1.47) and were engaged in (*M* = 5.35, *SD* = 0.90) the task more than they predicted (*M* = 3.31, *SD* = 1.58; *M* = 4.77, *SD* = 1.14), *ts* (49) = 4.68 and 3.67, *ps* < 0.05, *ds* = 0.66, and 0.52.These results provide initial evidence that participants underestimate their actual motivation during the task.

## Experiment 2

The purpose of Experiment 2 was to replicate the findings with a different task and with a between-subjects design where we manipulated the prediction and actual motivation as a between-subject factor. We used a between-subject design because there is possibility that making a prediction influenced actual motivation ratings in Experiment 1 (i.e., prediction effect). We aimed to replicate the underestimation effect observed in Experiment 1 after eliminating this prediction effect. In addition, for exploratory purposes, we manipulated task difficulty to address the possibility that the findings from Experiment 1 were caused by the relatively high task performance (i.e., participants generally felt that they were doing well, which might have increased their task engagement). Again, we expected that participants would show higher actual motivation (as measured by task enjoyment and engagement) than predicted motivation. We did not have a specific prediction about the effects of task difficulty.

## Method

### Participants

A total of 83 (73 female, *M*_*age*_ = 19.6) students at a UK university participated in the study. Participants were recruited by university SONA system and received course credit for participation. They were randomly assigned to one of four conditions in a factorial 2 (Prediction: predicted motivation and actual motivation) X 2 (Task Difficulty: easy and difficult) design. The majority of participants completed the experiment in a group session with two participants.

### Measures

We employed the same measures of task engagement (3 items) and task enjoyment (3 items) as Experiment 1 (Cronbanch’s αs = 0.73–0.89). For predicted motivation and actual motivation, the correlation between task engagement and task enjoyment was 0.50 (*p* < 0.001) and 0.48 (*p* = 0.001), respectively.

### Task

A total of 16 easy lists and 16 difficult lists of word-ordering task (one for the practice task and the other 15 for the main task) were used in a paper-and-pencil format. Each list comprised eight words. Participants were presented with a word list and had one minute to re-write the list in alphabetical order on their task sheet. Easy word lists typically included words that were simple to differentiate from each other: For example, they had different letters at the start of each word; *anterior*, *barracks* and *chewed*. Conversely, for the difficult word lists, the majority of words shared the same first and some subsequent letters, making them more difficult to differentiate; *hasten*, *hastily* and *hatched.*

### Procedure

On arriving in the lab room, participants were given instructions about the task. Participants then completed a one-minute practice trial of the word-ordering task, at the difficulty level they had been assigned, followed by self-reported motivation questions about the practice trial. In the main task, participants were first informed that they would go through 15 word lists and they would have one minute for each word list. Before the main task, participants in the predicted motivation condition were asked to make a prediction about their task engagement and task enjoyment after the main task. Participants in the actual motivation condition did not make a prediction before the main task, but rated their task engagement and task enjoyment after completing the main task.^4^

### Results and discussion

To examine whether the task difficulty manipulation was successful, a 2 (Prediction: predicted motivation and actual motivation) X 2 (Task Difficulty: easy and difficult) between-subjects ANOVA was conducted on task performance (i.e., the number of correctly sorted words). The main effect of Task Difficulty was significant, *F*(1, 79) = 143.6, *p* < 0.01, *η*_*G*_^2^ = 0.65, indicating that task performance in the Easy condition was higher (*M* = 7.44, *SD* = 0.74) than in the Difficult condition (*M* = 4.30, *SD* = 1.54). Neither the main effect of condition nor the interaction effect were statistically significant, *ps* = 0.05 and 0.86, respectively.

**Fig. 1:**
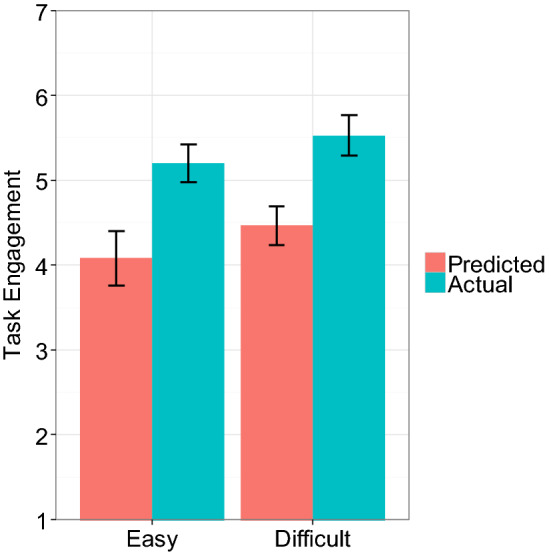
Task engagement as a function of Prediction and Task difficulty in Experiment 2. Error bars represent standard errors

To test our hypothesis, the same 2 X 2 ANOVA was conducted on task engagement and task enjoyment. Results showed, as illustrated in Fig. 1, that participants’ task engagement exhibited a significant main effect of Prediction, *F* (1, 79) = 18.06, *p* < 0.01, *η*_*G*_^2^ = 0.19, indicating that participants’ prediction about task engagement (for the Easy condition, *M* = 4.08, *SD* = 1.47; for the Difficult condition, *M* = 4.47, *SD* = 1.02) was lower than their actual task engagement (for the Easy condition, *M* = 5.20, *SD* = 1.00; for the Difficult condition, *M* = 5.53, *SD* = 1.12). Neither the main effect of Task Difficulty nor the interaction effect were significant, *ps* = 0.17 and 0.91, respectively. These findings replicated the results of Experiment 1. On the other hand, for task enjoyment, none of the effects were statistically significant, *η*_*G*_^2^ = 0.00–0.02, *ps* > 0.21 (Easy condition: Prediction, *M* = 3.78, *SD* = 1.24, Actual, *M* = 4.43, *SD* = 1.05, Difficult condition: Prediction, *M* = 3.95, *SD* = 1.16, Actual, *M* = 3.92, *SD* = 1.44). These results provided additional evidence that people underestimate task motivation, but this was observed only for task engagement, not for task enjoyment. We did not have a good idea of why this happened at the time of the experiment and we decided to see if the findings were robust in the following experiments.

## Experiment 3a

Experiments 1 and 2 showed that people become intrinsically motivated more than they predicted (especially in terms of task engagement) without performance-based extrinsic incentives. However, it is possible that these findings do not specifically reflect people’s tendency to underestimate the power of intrinsic motivation without performance-based extrinsic incentives, but simply indicate people’s general tendency to underestimate all types of motivated behaviors (see Scholer & Miele, 2016 for the importance of considering different types of motivation in examining metamotivational belief). To tease apart these two possibilities, Experiment 3 manipulated the provision of performance-based extrinsic rewards. If the previous results were caused, at least in part, by people’s underestimation of intrinsic motivation (without performance-based extrinsic incentives), the underestimation is expected to be reduced when people make a prediction about their motivated behavior which is contingent upon performance-based extrinsic incentives.

Experiment 3a also sought to replicate and extend the previous findings in two respects. First, we assessed participants’ motivation not only with self-reported questions, but also with a task that can objectively quantify task engagement. Second, as it is possible that inaccuracy of prediction may be a function of time and would not occur when participants predict motivation for a shorter task, we also manipulated the time duration of the task to explore whether and to what degree the accuracy of metamotivation changes as a function of task length.

We expected that, in the no reward condition, participants would again show higher actual motivation (as measured by task enjoyment and engagement) than predicted motivation. We also expected that this tendency would be reduced in the reward condition (i.e., we expected an interaction between the reward condition and prediction). We included time as an exploratory factor, but we thought that the underestimation effect in the no reward condition might be bigger as the task on time increases (i.e., an interaction between the reward condition, prediction, and time).

## Method

### Participants

A sample of 167 (86 female, *M*_*age*_ = 18.5) students at a Japanese university were recruited, after excluding two participants prior to data analysis (one for whom the experimenter incorrectly timed the experiment, and another who was disengaged throughout the experiment). Participants were randomly assigned to one of eight conditions in a factorial 2 (Reward: no reward and reward) X 4 (Time: 1 min, 5 min, 10 min, and 20 min) design. Motivation prediction was manipulated within subjects. The experiment was run individually in a lab room.

### Measures

We employed the same measures of task engagement (3 items) and task enjoyment (3 items) as Experiments 1 and 2 (Cronbanch’s αs = 0.82–0.89). These questions were rated on a 5-point scale in this experiment. For predicted motivation and actual motivation, the correlation between task engagement and task enjoyment was 0.44 (*p* < 0.001) and 0.43 (*p* = 0.001), respectively.

### Task

We used a self-regulatory task reported in Baumeister et al. (1998). Participants were provided with papers of academic articles written in English and were told to cross off all instances of the letter *e* within the time limit. For the practice task consisting of three trial runs (30 s per trial), we prepared three short paragraphs. For the main task (1 min, 5 min, 10 min, or 20 min), we prepared eight papers full of words. For both the practice trials and the main task, the number of words is so large that, when participants made a prediction, it is very unlikely that they took into account the possibility of finishing all the materials within the time limit.

The task was very simple and straightforward, meaning participants’ task engagement should directly influence the performance of the task. In other words, the performance of this task (i.e., the number of letters *e* crossed off within the time limit) can be considered to be linearly related to the degree of active engagement in the task. Accordingly, we used the number of letter *e*s crossed off as an alternative index of task engagement.

### Procedure

Participants were instructed about the task, and worked on three 30-s practice trials, followed by self-reported questions about the trials. For these practice trials, participants were provided with the feedback on how many letters participants correctly crossed off for each 30-s trial. Participants were then told about the time limit of the main task, depending on their experimental conditions (1 min, 5 min, 10 min or 20 min). Participants in the reward condition were further told that they would obtain 1 Japanese yen (approximately 1 cent) for each letter *e* that they correctly crossed off.

Before the main task, participants were asked to make a prediction about their task engagement by (1) rating their predicted task engagement and task enjoyment at the end of the experiment and (2) indicating how many letters they thought they would cross off within the given time limit. To facilitate the accurate calibration of their basic performance for the task, participants were allowed to look back at the feedback they obtained for the practice trials. After making the prediction, participants worked on the main task, and upon finishing the task, they rated their current task engagement and task enjoyment.

### Results and discussion

In the following analysis, one participant who made an unrealistically high prediction about task performance (more than 5*SD* above the mean across participants of the same task duration) was eliminated. To examine the effects of reward and task duration on the accuracy of metamotivation, we conducted a linear mixed-effects model (Murayama et al., 2014) with Prediction (effect coded; − 1 = actual motivation; 1 = predicted motivation), Reward (effect coded; − 1 = no reward; 1 = reward), Time (continuous variable; 1, 5, 10, and 20), and their two-way and three-way interactions as the predictors of task engagement, task enjoyment, and task performance. The analysis was conducted with Mplus (Muthen & Muthen, 2004). Participant intercepts were treated as a random effect. Participant slopes were not modelled because of a convergence problem.

**Fig. 2:**
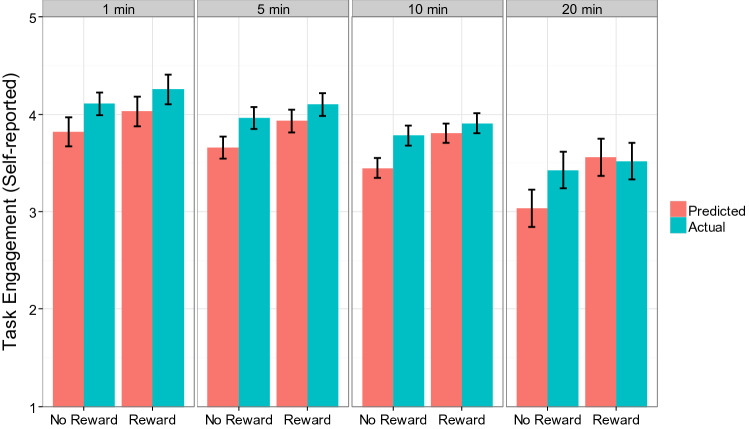
Self-reported task engagement as a function of Prediction, Reward, and Time in Experiment 3a (predicted values from the mixed effects model). Error bars represent standard errors, which were computed using the delta method

For task engagement, consistent with the previous experiments, the results revealed a significant main effect of Prediction, *B* = − 0.11 (standardized beta =− 0.18), *SE* = 0.03, *p* < 0.01, indicating that participants generally underestimated their self-reported task engagement for the main task. Importantly, this effect was qualified by the critical Prediction X Reward interaction, which was marginally significant, *B* = 0.054 (standardized beta = 0.085), *SE* = 0.03, *p* = 0.077. Simple main effect analysis showed that participants underestimated task engagement in the no reward condition, *B* = − 0.165 (standardized beta = − 0.263), *SE* = 0.04, *p* < 0.01, whereas this effect was not significant in the reward condition, *B* = − 0.057 (standardized beta = − 0.092), *SE* = 0.04, *p* = 0.20. To visualize the nature of the observed Prediction X Reward interaction effect and the other effects, Fig. 2 plotted the predicted values of task engagement for each condition. As is clear from the Fig. 2, overall, the underestimation of task engagement is observed in the no reward condition, but it is less clear in the reward condition.

For task enjoyment, consistent with task engagement, the results showed a significant main effect of Prediction, *B* = − 0.12 (standardized beta = − 0.19), *SE* = 0.03, *p* < 0.01, indicating that participants generally underestimated their self-reported task enjoyment; hence they enjoyed it more than they predicted they would. However, this effect was not qualified by higher-order interactions (*ps* > 0.21), indicating that the manipulation of reward did not have a significant influence on the underestimation of task enjoyment.

**Fig. 3:**
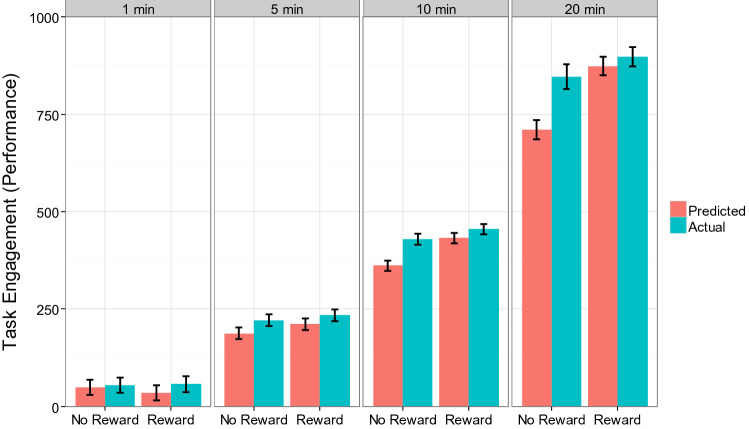
Task performance (objective measure of task engagement) as a function of Prediction, Reward, and Time in Experiment 3a (predicted values from the mixed effects model). Error bars represent standard errors, which were computed using the delta method

The same analysis was applied to task performance (i.e., objective measure of task engagement). Again, the results exhibited a significant main effect of Prediction, *B* = − 21.1 (standardized beta = − 0.07), *SE* = 6.1, *p* < 0.01, indicating that participants generally underestimated their task performance. Importantly, this effect was qualified by a significant Prediction X Reward X Time three-way interaction, *B* = 1.7 (standardized beta = 0.04), *SE* = 0.9, *p* < 0.05. Simple interaction analysis showed that, in the no reward condition, participants generally underestimated their task performance, *B* = − 30.2 (standardized beta = − 0.10), *SE* = 9.2, *p* < 0.01 and this underestimation was magnified as the duration of the task increased, *B* = − 3.4 (standardized beta = − 0.08), *SE* = 1.3, *p* < 0.01. However, in the reward condition, neither the overall tendency of underestimation, nor the change in the pattern as a function of Time was observed, *ps* > 0.15. To visualize the nature of this three-way interaction, Fig. 3 plots the predicted values of task performance. Consistent with the simple interaction analysis, the underestimation of motivation was generally observed in the no reward condition, especially when task duration is longer, but this pattern was not observed in the reward condition.

In sum, the findings provided strong evidence that participants underestimated motivation for both self-reported motivation and objective measure of motivation. Note that, unlike the previous study, the underestimation occurred for both task engagement and enjoyment. The results also seem to suggest that the tendency reduces when reward is promised for participants, but the results were not very clear (e.g., only marginally significant interaction for task engagement). Time seems to be a moderating factor of the underestimation effect (i.e., the effect is larger when time on task is longer) but the significant interaction effect was observed only for the objective measure of task engagement.

## Experiment 3b

The aim of Experiment 3b was to replicate the findings that people underestimate their task engagement even with an objective measurement of motivation (i.e., letters crossed off) in the no reward condition. To simplify the design, we only focused on the 5-min version of the previous experiment. Furthermore, we extended and examined the robustness of the prior study by additionally investigating the effects of prior warnings about the underestimation of motivation. Several studies have suggested that people’s metacognitive inaccuracy is rather persistent and resistant even after they receive verbal warnings (Yan et al., 2016). We were interested in whether metamotivational inaccuracy also shows this resistance to verbal warnings.

Like the previous experiments, we expected that participants would show higher actual motivation (as measured by task enjoyment and engagement) than predicted motivation (note that we did not have reward or time manipulations in this experiment). We did not have a specific prediction about the warnings, but given the previous findings that metacognitive inaccuracy is persistent (e.g., Yan et al., 2016), we thought that the underestimation effect might not be reduced even after the warning (i.e., no significant interaction effect between prediction and warning).

### Participants

A sample of sixty-four (forty-six female, age information not collected) university students at a Japanese university were recruited and received course credit for participation. Participants were randomly assigned to one of two conditions: a warning condition or a control condition. Motivation prediction was manipulated within subjects (i.e., the same participants were measured both on predicted and actual motivation with self-reported measures and objective index of task engagement). The experiment was conducted in a lab room.

### Measures

We employed the same self-reported measures of task engagement (3 items) and task enjoyment (3 items) as Experiment 3a (Cronbanch’s αs = 0.72–0.82). In addition to these measures, the experiment included 16 items which were prepared as filler items. In predicted motivation and actual motivation, the correlation between task engagement and task enjoyment was 0.34 (*p* = 0.009) and 0.33 (*p* = 0.009), respectively.

### Tasks

The task was identical with that used in Experiment 3a, expect that the current study only used the 5-min version in the no reward condition, and motivation prediction was manipulated within participants.

### Procedure

Participants were instructed about the task and worked on three 30-s practice trials. For these practices, participants were provided with feedback each time so that they could accurately calibrate their task performance (i.e., number of letter *e* crossed off) when making a prediction.

In the warning condition, participants were provided with the following warning before making the prediction: “Several recent studies, including Deci et al. (2015) and Murayama et al., (2016a, 2016b), have shown that participants tend to mispredict their motivation and performance for the task. Specifically, the studies have found that participants tend to rate their predicted motivation and performance lower than their actual motivation and performance after the task. Simply put, many people think that they would not be that motivated to work on the task before doing it, but in fact, they were more motivated than their prediction. Based on these findings, please try to predict as accurately as possible how you will feel after the task is completed.”

Before the main task, participants were asked to predict their motivation after the main task in terms of ratings of intrinsic motivation/task engagement and task performance (i.e., number of letter *e* crossed off). Participants then performed the main task. After the completion of this task, participants again rated their current task engagement and task enjoyment.

### Results and discussion

Prior to conducting the main data analysis, we excluded two participants due to procedural errors, and two other participants whose task performance was more than 2*SD* from the mean. As a result, there were 30 participants in each of the conditions.

A 2 (Prediction: predicted motivation and actual motivation) X 2 (Waring: warning and control) ANOVA was conducted on the self-reported task engagement and task enjoyment. Replicating the previous findings, the ANOVA for task engagement showed a significant main effect of Prediction, *F* (1, 58) = 13.15, *p* < 0.001, *η*_*G*_^2^ = 0.05, indicating that participants underestimated their task engagement (*M* = 3.80, *SD* = 0.81) in comparison to their actual task engagement (*M* = 4.12, *SD* = 0.66). Although task engagement was numerically higher in the warning condition (*M* = 4.12, *SD* = 0.78) than in control condition (*M* = 3.80, *SD* = 0.69), the main effect of warning did not reach significance, *F* (1, 58) = 3.73, *p* = 0.060, *η*_*G*_^2^ = 0.05. The interaction effect was also not significant, *F* (1, 58) = 0.14, *p* = 0.71, *η*_*G*_^2^ = 0.00. Analysis of task enjoyment also showed a significant main effect of Prediction, *F* (1, 58) = 15.27, *p* < 0.001, *η*_*G*_^2^ = 0.04. This result indicated that participants underestimated their task enjoyment (*M* = 3.12, *SD* = 0.76) in comparison to their actual task enjoyment (*M* = 3.43, *SD* = 0.86). The other main or interaction effects were not statistically significant, *η*_*G*_^2^ = 0.00-0.01, *ps* > 0.33.

To examine the difference between predicted and actual task performance (i.e., behavioral assessment of motivation), a 2 (Prediction: predicted task performance and actual task performance) X 2 (Warning: warning and control) ANOVA was conducted. The main effect of Prediction was significant, *F* (1, 58) = 58.87, *p* < 0.001, *η*_*G*_^2^ = 0.30, indicating that participants underestimated their task performance (*M* = 161.1, *SD* = 71.65) in comparison to their actual task performance (*M* = 231.1, *SD* = 27.40). The other main or interaction effects were not statistically significant, *η*_*G*_^2^ = 0.01–0.03, *ps* > 0.09.

The findings nicely replicated Experiment 3a—participants showed clear underestimation for both self-reported and objective measure of motivation. Consistent with the previous work on metacognition (e.g., Yan et al., 2016), the results were robust regardless of whether participants were warned about the possibility of underestimation.

## Experiments 4a and 4b

In Experiments 4a and 4b, we aimed to replicate the findings of Experiment 3a with different tasks. Here we manipulated prediction and reward (both between subjects). We again expected that participants would show higher actual motivation (as measured by task enjoyment and engagement) than predicted motivation in the no reward condition, but this effect would be reduced in the reward condition (i.e., we expected an interaction between the reward condition and prediction in both Experiments 4a and 4b).

### Participants

A total of 95 UK adults were recruited online from Scientific Prolific for Experiment 4a. An additional 101 UK adults were recruited online from the same platform in Experiment 4b. All participants were paid £3.34 as the baseline fee for their participation through Scientific Prolific. Note that participants received additional bonus (see Procedure). However, we excluded participants whose error rate was more than 20% prior to the main data analysis, resulting in 80 participants for Experiment 4a (27 female, *M*_*age*_ = 24.2) and 80 participants for Experiment 4b (35 females, *M*_*age*_ = 24.0). For each experiment, participants were randomly assigned to one of 2 (Prediction: predicted motivation and actual motivation) X 2 (Reward: no reward and reward) conditions. The experiments were run online and were part of a larger study examining the relationship between cognitive demand and effort avoidance (Kuratomi, Shigemasu, & Murayama, in prep). Motivation assessments were included in these experiments to specifically test the hypothesis of the current research.

### Measures

We employed the same measures of task engagement (3 items) and task enjoyment (3 items) as Experiment 3 (Cronbach’s αs = 0.59–0.92). For predicted motivation and actual motivation in Experiment 4a, the correlation between task engagement and task enjoyment was 0.51 (*p* < 0.001) and -0.14 (*p* = 0.397), respectively. For predicted motivation and actual motivation in Experiment 4b, the correlation between task engagement and task enjoyment was 0.48 (*p* = 0.002) and 0.26 (*p* = 0.105), respectively.

### Tasks

In Experiment 4a, participants worked on a flanker task and in Experiment 4b, participants worked on a number judgement task. In the flanker task, participants were presented with one the four letter-strings for 100 ms—NNNNN, ZZZZZ, ZZNZZ, NNZNN. Their task was to identify the central letter of the string (ignoring the other letters) as “N” or “Z” by pressing a button. In the number judgement task, participants were presented with a number between 1 and 9 (excluding 5) and their task was to make a judgement depending on the color of the number. Specially, when the number was in red, participants were required to judge whether the number was odd or even by pressing a key (i.e., parity judgement). When the number was in blue, participants were required to judge whether the number was smaller or larger than 5 (i.e., magnitude judgement). Key and color assignments were counterbalanced across participants. In both experiments, before seeing a stimulus, participants were presented with two different card decks and asked to select one of the decks. Unbeknownst to the participants, one card deck was associated with higher task demands (i.e., participants were likely to see a mixed-letter string or more frequent switch of the color or the number) than the other card deck. This experimental manipulation was made to address the main purpose of the larger research project that was irrelevant to the current research. For both tasks, there was one block of 400 trials and the task length was about 30 min.

### Procedure

Participants were first given instructions about the main task (flanker task in Experiment 4a or number judgement task in Experiment 4b) and completed the practice trials (20 trials for Experiment 4a and 30–60 trials for Experiment 4b depending on the task performance of the practice trials) and self-reported motivation questions about the practice trials. In the main task, participants were informed of the length of the task (30 min), and participants in the reward condition were further told that they would earn £ 0.01 for every 2 successful trials. Participants in the predicted motivation condition were then asked to make a prediction about their task engagement and task enjoyment after the main task. Conversely, participants in the actual motivation condition only rated their current task engagement and task enjoyment after finishing the main task. After both ratings were completed, participants in the reward condition were informed of the size of their upcoming bonus payment.

### Results and discussion

#### Experiment 4a (Flanker task)

Error rate for the task was generally low (7.3%), indicating that these participants were generally focused on the task.

**Fig. 4:**
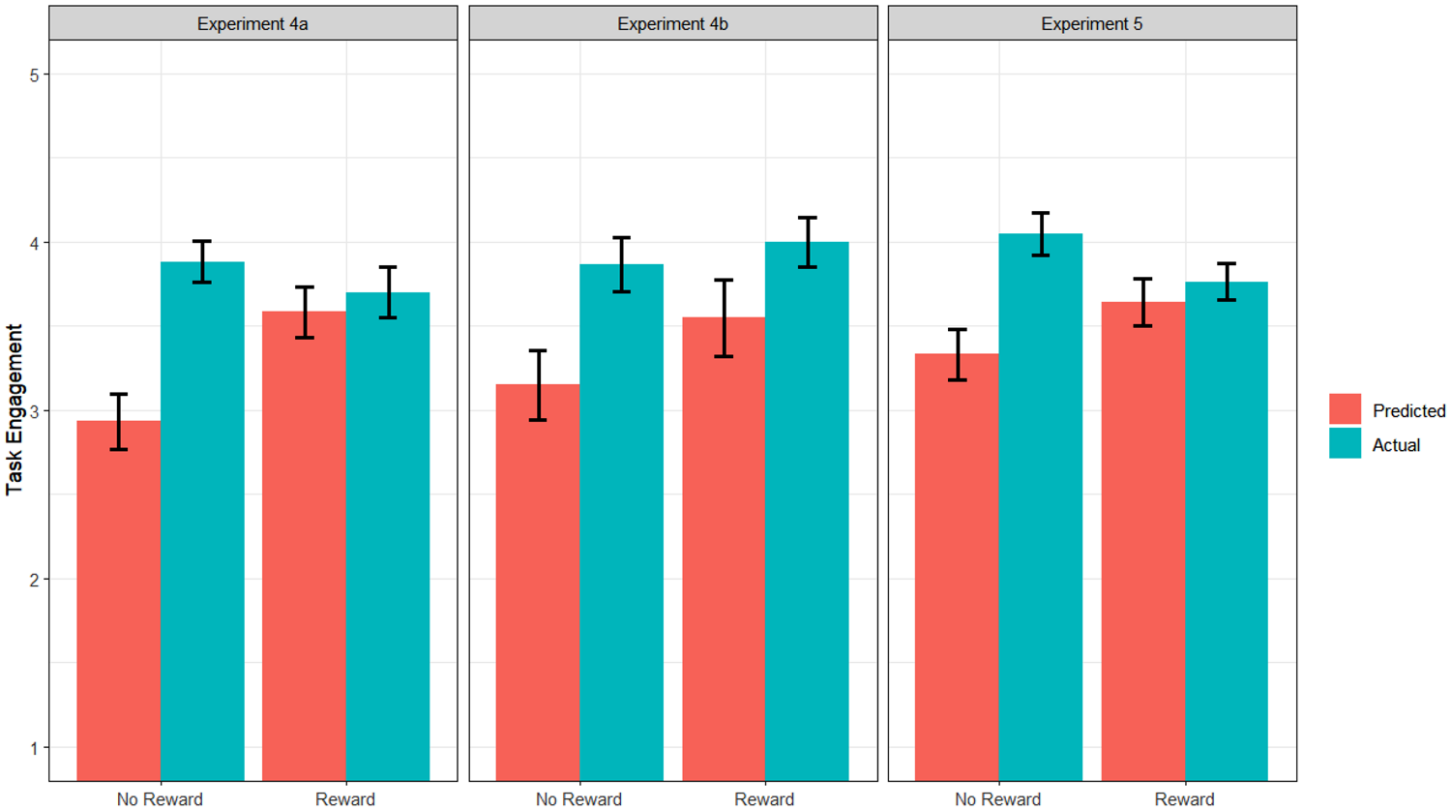
Task engagement as a function of Prediction and Reward in Experiment 4 and 5. Error bars represent standard errors

To test our hypothesis, the same 2 (Prediction: predicted motivation and actual motivation) X 2 (Reward: no reward and reward) conditions ANOVA for each experiment was conducted on task engagement and task enjoyment. Replicating the previous findings, results for task engagement showed a significant main effect of Prediction, *F* (1, 76) = 13.09, *p* < 0.001, *η*_*G*_^2^ = 0.15, indicating that participants underestimated their task engagement (*M* = 3.26, *SD* = 0.77) in comparison to their actual task engagement (*M* = 3.79, *SD* = 0.61). The main effect of the reward did not show a significant difference, *F* (1, 76) = 2.51, *p* = 0.12, *η*_*G*_^2^ = 0.03. Critically, a significant Prediction X Reward interaction, *F* (1, 76) = 7.99, *p* < 0.01, *η*_*G*_^2^ = 0.10, was obtained. As can be seen in Fig. 4, whereas participants significantly underestimated task engagement in the no reward condition, *F* (1, 76) = 20.8, *p* < 0.001, *η*_*G*_^2^ = 0.21, that underestimation was not observed in the reward condition, and indeed it was no longer significant, *F* (1, 76) = 0.31, *p* = 0.58, *η*_*G*_^2^ = 0.00. These results indicate that participants underestimate their motivation especially in the absence of performance-based extrinsic incentives.

Results for task enjoyment in Experiment 4a also showed a significant main effect of Prediction, *F* (1, 76) = 16.58, *p* < 0.001, *η*_*G*_^2^ = 0.18. These results mirror the results of the task engagement, suggesting that participants underestimated their task enjoyment (*M* = 2.11, *SD* = 1.00) in comparison to their actual task enjoyment (*M* = 2.92, *SD* = 1.02). The main effect of Reward was also statistically significant, *F* (1, 76) = 24.95, *p* < 0.001, *η*_*G*_^2^ = 0.25. However, unlike task engagement (and consistent with Experiment 3), the critical Prediction X Reward interaction was not statistically significant, *F* (1, 76) = 0.30, *p* = 0.59, *η*_*G*_^2^ = 0.00.

#### Experiment 4b (Task switch)

Error rate for the task was generally low (10.4%), indicating that these participants were generally focused on the task.

To test our hypothesis, the same ANOVA with Experiment 4a was conducted on the task engagement and task enjoyment. Replicating the previous findings, results for task engagement showed a significant main effect of Prediction, *F* (1, 76) = 9.61, *p* < 0.01, *η*_*G*_^2^ = 0.11, indicating that participants underestimated their task engagement (*M* = 3.35, *SD* = 0.98) in comparison to their actual task engagement (*M* = 3.93, *SD* = 0.68). The main effect of the reward was not significant, *F* (1, 76) = 2.01, *p* = 0.16, *η*_*G*_^2^ = 0.03, and neither was the Prediction X Reward interaction, *F* (1, 76) = 0.50, *p* = 0.48, *η*_*G*_^2^ = 0.01.

Results for task enjoyment in Experiment 4b also showed a significant main effect of Prediction, *F* (1, 76) = 7.71, *p* < 0.01, *η*_*G*_^2^ = 0.09. These results mirror the results for task engagement, suggesting that participants underestimated their task enjoyment (*M* = 2.00, *SD* = 0.84) in comparison to their actual task enjoyment (*M* = 2.55, *SD* = 0.93). Neither the main effect of Reward nor the interaction effect were statistically significant, *ps* > 0.13.

In sum, there are two main findings. First, we again robustly obtained the underestimation effect for both task enjoyment and engagement. Participants tended to underestimate their actual motivation. Second, the expected Reward x Task interaction effect was observed only for the flanker task (and only for task engagement), but not the number judgement task. Given the inconsistent results between the experiments, we conducted a replication experiment that aims to address whether the effect is reliable or not.

## Replication of reward effect (Experiment 5)

In Experiments 3a, 4a, and 4b, we showed that people underestimated how engaging the task would be without performance-based incentives; but this effect was weaker when participants were provided with performance-based extrinsic incentives. However, in Experiment 3a, the critical Prediction X Reward interaction was only marginally significant (*p* = 0.077; although a similar effect was observed in task performance). In Experiment 4b, the Prediction X Reward interaction was not significant. To investigate the robustness of our finding, we conducted an exact replication of Experiment 4a.^5^ Experiment 4a was selected because it showed the largest effect size for the Prediction X Reward interaction effect. We thus expected to observe a Prediction X Reward interaction effect.

### Participants

In Experiment 4a, the effect size of the critical interaction effect between prediction and reward was 0.32 in Cohen’s *f* metric. Using this effect size estimate, 79 participants were required to achieve a statistical power of 80%. With this number in mind, we collected as much data as possible within budgetary limits. As a result, 127 participants were recruited for this replication study from Scientific Prolific, and after applying the same exclusion criteria as with Experiment 4a (determined a priori), 106 participants were included in the main data analysis (64 female, *M*_*age*_ = 25.3). The experiment was conducted online and participants were randomly assigned to one of the 2 (Prediction: predicted motivation and actual motivation) X 2 (Reward: no reward and reward) conditions. All participants were paid £3.34 as the baseline fee for their participation through Scientific Prolific. Note that participants received an additional bonus.

### Measures

We employed the same measures of task engagement (3 items) and task enjoyment (3 items) as Experiment 3 (Cronbach’s αs = 0.43–0.88). For predicted motivation and actual motivation, the correlation between task engagement and task enjoyment was 0.28 (*p* = 0.045) and 0.39 (*p* = 0.003), respectively.

### Tasks and procedure

The experiment employed the same flanker task used in Experiment 4a. The procedure was also identical to Experiment 4a.

### Results and discussion

Error rate for the task was generally low (7.6%), indicating that participants were largely focused on the task.

To test our hypothesis, 2 (Prediction: predicted motivation and actual motivation) X 2 (Reward: no reward and reward) ANOVA was conducted on the engagement and task enjoyment. Replicating our main finding, task engagement demonstrated a significant main effect of Prediction, *F* (1, 102) = 10.20, *p* < 0.01, *η*_*G*_^2^ = 0.09, suggesting that participants again underestimated their task engagement when making a prediction (*M* = 3.50, *SD* = 0.74) in comparison to their actual task engagement (*M* = 3.90, *SD* = 0.62). Importantly, this main effect was qualified by a significant Prediction X Reward interaction, *F* (1, 102) = 5.21, *p* < 0.05, *η*_*G*_^2^ = 0.05. As indicated in Fig. 4, participants underestimated task engagement in the no reward condition, *F* (1, 102) = 14.16, *p* < 0.01, *η*_*G*_^2^ = 0.12, but this underestimation was substantially diminished in the reward condition, *F* (1, 102) = 0.44, *p* = 0.51, *η*_*G*_^2^ = 0.00. The main effect of reward was not statistically significant, *F* (1, 102) = 0.01, *p* = 0.93, *η*_*G*_^2^ = 0.00.

Unlike the previous experiment, task enjoyment also showed a similar pattern. Specifically, 2 X 2 ANOVA showed a significant main effect of Prediction, *F* (1, 102) = 15.90, *p* < 0.01, *η*_*G*_^2^ = 0.13, suggesting an overall underestimation of task enjoyment (*M* = 2.34, *SD* = 0.86 for their predicted task enjoyment; *M* = 2.99, *SD* = 0.97 for their actual task enjoyment). The main effect of reward was also significant, *F* (1, 102) = 6.13, *p* < 0.05, *η*_*G*_^2^ = 0.0.06. Critically, the Prediction X Reward interaction also exhibited a significant effect, *F* (1, 102) = 4.80, *p* < 0.05, *η*_*G*_^2^ = 0.04. Simple main effect analyses revealed that participants in the no reward condition significantly underestimated task enjoyment, *F* (1, 102) = 18.01, *p* < 0.01, *η*_*G*_^2^ = 0.15 (*M* = 1.90; *SD* = 0.66 for predicted task enjoyment; *M* = 2.96, *SD* = 0.92 for the actual task enjoyment) but this was not observed in the reward condition, *F* (1, 102) = 1.72, *p* = 0.19, *η*_*G*_^2^ = 0.02 (*M* = 2.70; *SD* = 0.83 for predicted task enjoyment; *M* = 3.01, *SD* = 1.04 for actual task enjoyment).

These results indicate again the robustness of the underestimation effect. Importantly, we also confirmed that the Prediction x Reward interaction effect is a robust phenomenon, for both aspects of motivation (i.e., task enjoyment and engagement). Specifically, participants exhibited underestimation of motivation with no reward, but this effect was not significant when reward was contingent on the task.

### Supplementary exploratory analysis for all experiments

To further understand the nature of underestimation, we conducted the following two analyses for all the experiments. First, we computed the correlation between task performance of the main task (e.g., the number of words generated in Experiment 1, error rate in Experiment 4a, 4b, and 5) for predicted motivation (enjoyment and engagement), actual motivation, and the difference between them (i.e., the extent of overestimation; only for the studies with a within-subjects design). We computed the correlation for each condition separately. The results are summarized in Table S1 in the supplementary online materials. The results showed seven significant correlations out of 98. Note that three of the significant correlations were observed for predicted motivation, which was rated before performing the task. These findings indicate that there is generally little evidence that task performance was related to the inaccuracy of metamotivation.

Second, recall that all the experiments, expect Experiment 3b, included a practice trial so that participants could get a feel of the task. This allowed us to compare actual motivation ratings for the practice trials and for the main task, and these results are summarized in Table S2. The results suggested that motivation significantly decreased from the practice trial to the main task. This is not surprising, as we intentionally chose monotonic, rather boring tasks for the study. However, interestingly, this was the case especially for enjoyment (10 significant differences out of 17 comparisons), rather than engagement (2 significant differences out of 17 comparisons). Although speculative, these results may potentially explain why task enjoyment did not consistently show the underestimation effect in comparison to task engagement. Specifically, there was a dissociation between the change of task enjoyment and task engagement—participants were able to keep their engagement relatively high while enjoyment went down. As a result, the underestimation effect was observed mainly for task engagement.

## General discussion

Across seven experiments using various tasks with different populations from different countries, we consistently observed that participants underestimated the extent to which they would be motivated to complete a task without performance-based extrinsic incentives. Specifically, participants were engaged in the task more actively without performance-based incentives than they predicted. These results demonstrate the inaccuracy of our metacognition about our ability to regulate and sustain motivation without extrinsic incentives—people can upregulate their motivation without extrinsic incentives more than they believe.

In the literature of metacognition, researchers have repeatedly found that we make inaccurate predictions about our future learning (Kornell & Bjork, 2008; Murayama et al., 2016a, 2016b; Rhodes & Castel, 2008; Soderstrom & Bjork, 2015). The literature of affective forecasting also shows that people routinely mispredict their future emotion and well-being (Wilson & Gilbert, 2005; see also Loewenstein et al., 2001; Schwarz, 2015). Our research expanded those findings, showing that such metacognitive inaccuracy is also observed in people’s predictions or beliefs about motivation (i.e., metamotivation).

Why is inaccurate metacognition a problem? As people’s decision making is largely based on their metacognitive belief about what is right, inaccurate metacognition may lead to suboptimal decision making. In fact, previous studies have showed that inaccurate metacognition inadvertently promotes the use of maladaptive learning strategies when people study (Dunlosky et al., 2013). Other studies also revealed that misprediction of future affect makes people fail to maximize their happiness (e.g., Gilbert & Ebert, 2002). In the context of metamotivation, Hatano et al. (2022) showed that people underestimate their enjoyment and engagement when asked to sit in a room alone and just think for 20 min. Importantly, because of this underestimation, when participants had an option between surfing the internet or just thinking, they tended to choose the former, thereby missing the opportunity to enjoy the thinking activities. As the current research found that people underestimate their own motivation without extrinsic incentives, the logical next step for future research is to examine the impact of such inaccurate metamotivation on people’s actual self- or other- motivating behaviors. For example, based on our findings that people underestimate their motivation in the absence of extrinsic incentives, it is possible that people may prefer relying on extrinsic incentives more than necessary to motivate themselves or others.

Another important step is to examine the mechanisms underlying this underestimation effect. There are several possibilities. For example, perhaps participants underestimated their actual intrinsic motivation because they were unable to appreciate their capability to generate strategies to make the task engaging (Sansone et al., 1992). If this is the case, future studies should examine what kind of spontaneous motivation regulation strategies people use when faced with a boring task. It is also possible that people did not appreciate the power of automation; that as they became more efficient at completing the task, the task would become less demanding. Perhaps participants were not able to understand that the physical and mental costs that they perceived in the practice trials would diminish over time. Another likely mechanism is that people overlooked the potential rewarding feelings that they acquired by becoming proficient in the task. Critically, these explanations are not mutually exclusive and it is possible that they jointly result in the underestimation effect. Future studies should systematically manipulate task properties to examine how and when people underestimate task engagement without performance-based extrinsic incentives.

It is worth noting that we consistently found underestimation of motivation for task engagement, but results were less consistent for task enjoyment. This observation suggests that our findings do not simply reflect the inaccuracy of the affective forecasting (Wilson & Gilbert, 2005) and cannot be fully explained by the factors underlying affective forecasting (e.g., emotional adaptation; Ubel et al., 2005). Research on interest argues that task engagement and task enjoyment are essential components of interest but represent different phases of interest development (Hidi & Renninger, 2006; Sansone & Thoman, 2005). Specifically, the literature suggests that initial (often extrinsic) task engagement precedes the development of task enjoyment, which requires more internalization of task values (see also Renninger & Hidi, 2002). As participants worked on the tasks for only up to 30 min in the current experiments, and participants were not provided with sufficient reasons to work on the task, it is likely that they did not develop interest to the extent they could internalize and enjoy the task. This is also consistent with our observation of the exploratory analysis that enjoyment in the main task was significantly decreased from practice trial while task engagement was relatively maintained. Although we did not specifically predict the different pattern of results between task engagement and task enjoyment, the current findings provided some evidence for the importance of distinguishing these elements of task interest (see also Goldsmith & Dhar, 2013).

As indicated in the introduction, several previous studies have examined people’s metamotivational belief on extrinsic incentives, and suggested that people tend to overestimate the motivational power of extrinsic incentives (Heath, 1999; Miller & Ratner, 1998; Woolley & Fishbach, 2015). Although the present study was not specifically designed to examine the accuracy of the motivational power of extrinsic incentives, a close scrutiny of the results exhibits a pattern consistent with these interpretations. That is, in Experiments 3a, 4, and the replication experiment, the predicted motivation substantially increased from the no-reward condition to the reward condition but the actual increase in motivation was much smaller or rewards even slightly decreased motivation (see Figs. 2–4). This pattern was supported by the statistically significant Prediction X Reward interactions reported in the results (see Woolley & Fishbach, 2015 for similar pattern of results). These observations indicate that people overestimate the positive effects of extrinsic rewards on motivation. This finding poses an interesting contrast with the underestimation of the power of intrinsic motivation.

In fact, the current findings on the effects of extrinsic incentives are somewhat consistent and complementary with the findings reported by Murayama et al., (2016a, 2016b). In Murayama et al., (2016a, 2016b), participants read the procedure of an experiment that empirically showed the “undermining effect”—a phenomenon where performance-contingent monetary rewards decreased voluntary engagement in an enjoyable task after performance-contingent rewards are no longer relevant (Murayama et al., 2010). After reading the procedure, participants made a prediction about the result—namely, whether their engagement would increase or decrease in comparison to a control group (or there would be no difference from the control group). The majority of participants (falsely) predicted that performance-contingent rewards would increase motivation even after performance-contingent rewards are no longer relevant with greater confidence. While there are several notable differences (e.g., the current study used boring tasks; we did not found decreased motivation in the reward condition) and our study is not designed to specifically test the effects of extrinsic incentives, our findings and results from Murayama et al., (2016a, 2016b) jointly make a strong case that people seem to believe that performance-based incentives would be effective to motivate people even in a situation where that is not the case.

A few limitations should be noted. First, while we obtained evidence for the underestimation effect with various boring tasks, the choice of the tasks was arbitrary and we still do not know whether the findings can be generalized to other types of tasks. As noted earlier, we should seek to understand the underlying mechanisms, which will help us to understand the extent to which our findings are generalizable. Second, participants were all young adults. Some previous studies have noted developmental differences in metacognitive accuracy (Palmer et al., 2014; Weil et al., 2013), and future research should examine whether our results are applicable to different age groups. Third, we only examined two aspects of motivation—engagement and enjoyment—but motivation is a multifaceted construct that consists of various elements (see Tamura et al., 2022). Future studies should examine the accuracy of metamotivation focusing on other elements in motivation. Finally, task enjoyment and engagement scales, which were taken from a previous study (Elliot & Harackiewicz, 1996) often showed relatively low Cronbach’s αs. While this may be perceived as problematic, we have consistent results across the experiments. In addition, these task enjoyment and engagement scales also showed reliable effects in a separate study on metamotivation (Hatano et al., 2022). However, it is worth examining the robustness of our findings using different scales assessing the same constructs.

Over the past decades, a vast amount of research has been conducted to examine the nature, determinants, and consequences of intrinsic motivation, cultivating and advancing our understanding of human motivated behavior (Ryan & Deci, 2017). A substantial body of research in cognitive psychology has also revealed that our metacognitive belief about cognitive process (e.g., memory) is often inaccurate, endangering our self-regulated behavior (Bjork et al., 2013). However, despite the prominence of both lines of work, and despite its critical role in applied settings such as education, organization, and policy making, surprisingly little research has integrated these perspectives, examining the metacognitive accuracy of intrinsic motivation. We hope that the current findings open a new avenue for a number of future research programs, elucidating more detailed characteristics of our belief in how intrinsically motivated we are.

## Supplementary Information

Below is the link to the electronic supplementary material.

Supplementary file1 (PDF 87 kb)

## Footnotes

1 We focused on these two aspects of motivation, as they are two related but distinct essential components of motivation --- enjoyment represents affective component whereas engagement represents the behavioral component of motivation (see also Hatano et al., 2022 for a similar distinction). Enjoyment is also a core component of intrinsic motivation (Deci & Ryan, 1985).

2 In all experiments participants received some form of compensation (e.g., money, course credit) for their participation. However, these compensations were made for their participation only (except for reward conditions in Experiments 4 and 5); compensations were not contingent upon the motivation or the quality of performance for the task.

3 Data from all experiments can be obtained in Open Science Framework (https://osf.io/pjq8m/). We also included other measures for exploratory purpose in Experiments 2 and 3 but task engagement and task enjoyment are the only measures that were commonly used across all experiments.

4 In Experiments 2 and 4a/4b (and the Replication Experiment), participants in the Prediction condition actually did the main task following the prediction, and rated their motivation after finishing the main task. This is because we needed to ensure that participants spent a certain amount of time on the experiment to justify the advertised compensation. The results of these measures (i.e., the analysis of predicted motivation and actual motivation focusing only on the participants in the predicted motivation condition) were consistent with the main findings reported in this paper, but we report the results of the comparison between predicted and actual motivation conditions (i.e., between-subject comparison), because this comparison is not contaminated by potential prediction effect (i.e., making a prediction may distort the ratings of actual motivation after the main task) and thus provides more accurate causal estimates. However, interested readers can re-analyze the data uploaded on the Open Science Framework (https://osf.io/pjq8m/).

5 This is the only exact replication study that we conducted.
