# Supplementary material for: People underestimate their capability to motivate themselves without performance-based extrinsic incentives
Source: Motiv Emot. 2022 Dec 28;47(4):509–23. doi: 10.1007/s11031-022-09996-5 (PMC10328886; doi:10.1007/s11031-022-09996-5)
Supplement: Supplementary file 1 — Supplementary file1 (PDF 87 kb) [file 11031_2022_9996_MOESM1_ESM.pdf]

Table 1. Correlation table for task performance.

		Predicted		Actual		Difference	
		**Enjoyment**	**Engagement**	**Enjoyment**	**Engagement**	**Enjoyment**	**Engagement**
**Experiment 1** (n = 50)		-.12	.05	.00	.21	.11	.15
**Experiment 2**							
Easy (Predicted = 21, Actual = 20)		.43 +	.46 *	.07	.14	-	-
Difficult (Predicted = 20, Actual = 22)		.10	.02	.14	.23	-	-
**Experiment 3a**							
1 min	Reward (n = 21)	-.38 +	-.09	-.19	.06	.25	.25
	Control (n = 22)	-.23	-.19	-.02	-.23	.20	-.05
5 min	Reward (n = 21)	-.08	.20	-.18	-.18	-.16	-.37
	Control (n = 21)	-.48 *	-.50 *	-.26	-.24	.23	.32
10 min	Reward (n = 20)	.02	-.10	-.02	-.26	-.04	-.16
	Control (n = 20)	.24	.15	.40 +	.47 *	.14	.49 *
20 min	Reward (n = 21)	.26	.03	.47 *	.35	.26	.33
	Control (n = 21)	.21	-.05	-.29	-.36	-.39 +	-.33
**Experiment 3b**							
Alert (n = 30)		-.10	.22	-.06	.14	.06	-.11
Control (n = 30)		.07	-.15	.26	.00	.26	.19
**Experiment 4a**							
Reward (Predicted = 20, Actural = 20)		-.15	.06	.13	.09	-	-
Control (Predicted = 20, Actural = 20)		-.25	-.39 +	.34	-.42 +	-	-
**Experiment 4b**							
Reward (Predicted = 20, Actural = 20)		-.31	.05	.05	.16	-	-
Control (Predicted = 20, Actural = 20)		-.18	-.11	.15	-.60 **	-	-
**Experiment 5**							
Reward (Predicted = 28, Actural = 28)		-.21	-.29	.16	.09	-	-
Control (Predicted = 27, Actural = 23)		.28	.12	.19	-.05	-	-

Note. Task performance is the average number of words generated per trial in Experiment1, correct response in Experiment 2, the number of erasures in Experiment 3, and error rate in Experiment 4 and 5. ** $p < .01$, * $p < .05$, + $p < .10$

Table 2. Comparison of post-practice and actual motivation in each experiment.

		Enjoyment					Engagement				
		**Practice**	**Actual**	***t***	***df***	***p***	**Practice**	**Actual**	***t***	***df***	***p***
**Experiment 1**		4.83 (1.22)	4.15 (1.47)	3.96	49	< .001	5.13 (0.92)	5.35 (0.90)	-1.48	49	.15
**Experiment 2**											
Easy		4.18 (1.14)	4.10 (1.19)	0.54	40	.59	4.88 (1.05)	4.63 (1.37)	1.31	40	.20
Difficult		4.07 (1.17)	3.94 (1.30)	0.95	41	.35	5.29 (1.03)	5.02 (1.19)	1.62	41	.11
**Experiment 3a**											
1 min	Reward	3.63 (0.84)	3.63 (0.78)	0.00	20	.99	3.89 (0.76)	4.06 (0.98)	-1.01	20	.33
	Control	3.55 (0.79)	3.65 (0.96)	-0.69	21	.50	3.86 (0.82)	4.12 (0.83)	-2.11	21	.05
5 min	Reward	3.89 (0.53)	3.49 (0.82)	2.93	20	.01	4.13 (0.79)	4.16 (0.80)	-0.13	20	.90
	Control	3.70 (0.89)	3.48 (0.83)	1.08	20	.29	4.08 (0.72)	4.21 (0.83)	-0.98	20	.34
10 min	Reward	3.75 (0.59)	3.54 (0.79)	1.45	20	.16	3.87 (0.88)	4.19 (0.98)	-1.79	20	.09
	Control	3.51 (0.94)	3.10 (0.84)	2.81	20	.01	3.81 (0.83)	3.40 (0.90)	1.78	20	.09
20 min	Reward	3.55 (0.75)	3.12 (1.03)	2.76	19	.01	3.67 (0.73)	3.37 (0.88)	1.94	19	.07
	Control	3.42 (0.79)	2.95 (0.91)	2.72	19	.01	3.98 (0.83)	3.57 (1.05)	2.12	19	.05
**Experiment 3b**		-	-		-		-	-		-	
**Experiment 4a**											
Reward		3.40 (1.01)	3.21 (1.12)	1.59	39	.12	3.75 (0.66)	3.75 (0.76)	0.00	39	.99
Control		2.85 (0.82)	2.26 (0.83)	4.36	39	< .001	3.72 (0.58)	3.74 (0.59)	-0.23	39	.82
**Experiment 4b**											
Reward		2.97 (1.02)	2.53 (1.02)	2.99	39	.01	3.72 (0.66)	3.83 (0.78)	-0.72	39	.48
Control		3.04 (0.94)	2.22 (0.93)	6.74	39	< .001	3.99 (0.66)	3.81 (0.76)	1.48	39	.15
**Experiment 5**											
Reward		3.11 (1.01)	2.52 (0.95)	4.57	55	< .001	3.60 (0.63)	3.70 (0.66)	-1.25	55	.22
Control		2.95 (1.00)	2.14 (0.96)	6.46	49	< .001	3.75 (0.61)	3.72 (0.77)	0.28	49	.78

Note. The Practice and Actual columns represent means (*SD*) of motivation.
